# Trends in Medicare Spending on Oral Drugs for Chronic Lymphocytic Leukemia From 2014 to 2020

**DOI:** 10.1001/jamanetworkopen.2023.7467

**Published:** 2023-04-07

**Authors:** Edward R. Scheffer Cliff, Aaron S. Kesselheim, Benjamin N. Rome, William B. Feldman

**Affiliations:** 1 Program on Regulation, Therapeutics and Law, Division of Pharmacoepidemiology and Pharmacoeconomics, Department of Medicine, Brigham and Women’s Hospital, Boston, Massachusetts; 2 Harvard Medical School, Boston, Massachusetts

## Abstract

This cross-sectional study analyzes trends in Medicare Part D use and spending on oral-targeted drugs for chronic lymphocytic leukemia from 2014 to 2020.

## Introduction

The treatment of chronic lymphocytic leukemia (CLL), the most common adult leukemia, has changed considerably in recent years. In 2013, the US Food and Drug Administration approved ibrutinib (Imbruvica), an oral inhibitor of Bruton tyrosine kinase (BTKi) that is both more effective and safer than previous standard-of-care chemotherapy.^1^ Ibrutinib is one of the most-prescribed oral cancer therapies. Among the top-selling drugs in the US, ibrutinib accounted for more than $2.8 billion in annual net Medicare spending by 2020.^2^

Subsequently, BTKis with fewer adverse effects, such as acalabrutinib, were approved for CLL, as was venetoclax, the first-in-class B-cell lymphoma-2 inhibitor, which offers additional clinical advantages such as time-limited therapy and the potential for complete remission.^1,3^ In 2014, phosphatidylinositol-3 kinase inhibitors were also approved for CLL, although they are less effective and more toxic and, thus, usually reserved for multiply relapsed patients.^1^

We hypothesized that the emergence of multiple oral-targeted drugs for CLL may lower costs due to competition and affect overall spending on ibrutinib. In this cross-sectional study, we analyzed trends in Medicare Part D use and spending on these drugs from 2014 to 2020.

## Methods

We used publicly available Medicare Part D data to determine annual spending on oral CLL drugs, the number of beneficiaries who received these drugs, and the average spending per 30-day fill.^4^ We estimated net spending after rebates using 4-quarter rolling average non-Medicaid rebates from SSR Health and adjusted spending to 2020 US dollars according to the consumer price index for all urban consumers. A sensitivity analysis was performed to evaluate net price trajectories beyond Medicare to assess whether mandatory coverage requirements of oncology products in Part D might be associated with trends. Microsoft Excel version 16.70 (Microsoft) was used to conduct the data analysis from March 2022 to October 2022. This study was not submitted for institutional review board approval and did not require informed consent because it used public, nonidentifiable data and did not constitute human participants research, in accordance with 45 CFR §46.

## Results

**Figure 1.**
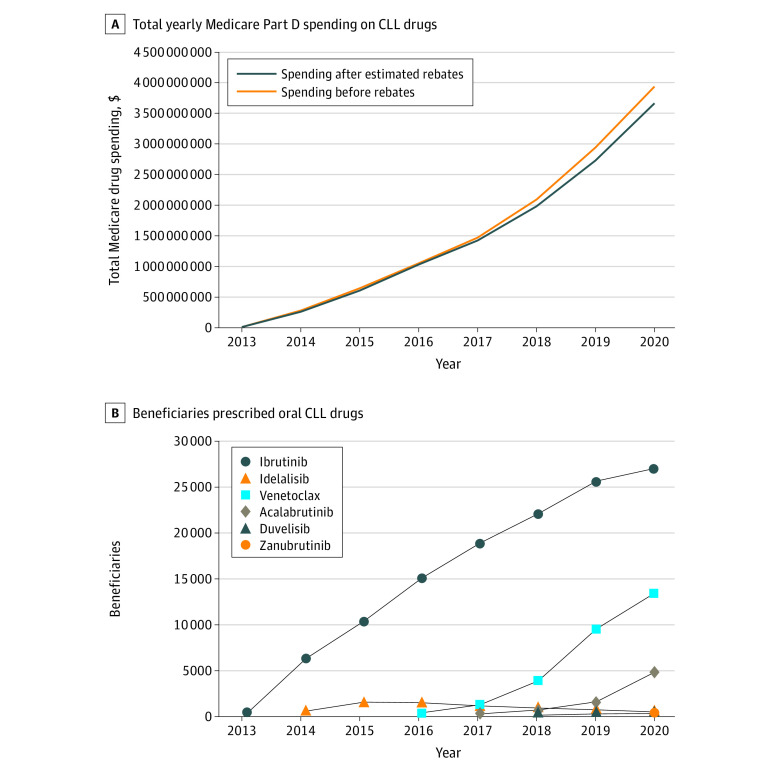
Growth in Spending and Prescribing of Oral Chronic Lymphocytic Leukemia Drugs A, Graph shows total yearly Medicare Part D spending on oral chronic lymphocytic leukemia drugs in millions of US dollars before and after estimated rebates. B, Graph shows number of Medicare beneficiaries prescribed oral CLL drugs each year.

Six oral medications were included in the study: 3 BTKis (ibrutinib, acalabrutinib, and zanubrutinib), 2 phosphatidylinositol-3 kinase inhibitors (idelalisib and duvelisib), and 1 B-cell lymphoma-2 inhibitor (venetoclax). From 2014 to 2020, annual net Medicare spending on these 6 drugs across all indications increased from $254 million to $3.7 billion (Figure 1). In 2014, 6180 beneficiaries received ibrutinib, increasing to 26 847 beneficiaries in 2020. Spending on ibrutinib constituted 77% of the total Medicare spending on these 6 drugs in 2020.

**Figure 2.**
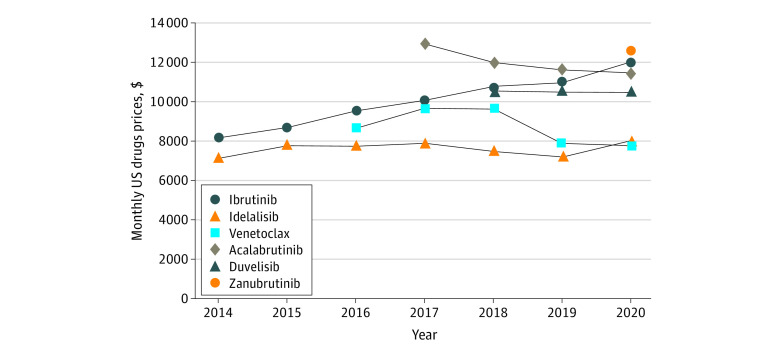
Medicare Spending per 30-Day Supply of Chronic Lymphocytic Leukemia (CLL) Drugs Graph shows Medicare spending per 30-day supply of each oral chronic lymphocytic leukemia drug in US dollars after adjustment for estimated rebates and inflation. Prices for 2013 were excluded because of the low sample size.

Estimated net spending per 30-day supply of ibrutinib increased 46%, from $8206 in 2014 to $11 980 in 2020, despite market entry of venetoclax in 2016 (2020 30-day fill price, $7787), acalabrutinib in 2017 ($11 428) and zanubrutinib in 2020 ($12 521) (Figure 2). A sensitivity analysis of net prices revealed a similar trend outside Medicare. Unlike ibrutinib, net spending per 30-day supply of other oral targeted drugs generally did not increase over time, and some drug prices decreased slightly.

## Discussion

As shown in this cross-sectional study, Medicare costs for a 30-day supply of ibrutinib, the first oral CLL drug, increased markedly from 2014 to 2020, despite competition from other drugs, some of which were less expensive than ibrutinib. The inability of brand to brand competition to lower prices has been observed in other contexts, particularly among cancer drugs.^5,6^ Brand to brand competition may have been ineffective at lowering Medicare costs due to lags between approval and change in prescriber practices, constraints on payers’ ability to effectively use formularies to negotiate prices, and financial incentives that can encourage intermediaries such as pharmacy benefit managers to accept high prices.^5^

Despite increased within-class and between-class competition from drugs with better clinical profiles, both the cost and the rate of prescribing ibrutinib increased markedly. One limitation of this study is that Medicare does not report spending by indication, so we cannot know what proportion of this spending is for CLL vs for other B-cell lymphomas. Further research is needed to understand why oncologists have not embraced clinically superior options for CLL being sold at prices similar to, if not lower than, ibrutinib.

## Notes

Supplement.
Data Sharing Statement

## References

[1] Mato AR, Davids MS, Sharman J, . Recognizing unmet need in the era of targeted therapy for CLL/SLL: “what’s past is prologue” (Shakespeare). Clin Cancer Res. 2022;28(4):603-608. doi:10.1158/1078-0432.CCR-21-1237 34789482 PMC9253788

[2] Mikulic M. Leading cancer drugs worldwide by revenue in 2020. Accessed October 24, 2022. https://www.statista.com/statistics/288538/top-cancer-drugs-based-on-revenue/

[3] Eyre TA, Lamanna N, Roeker LE, . Comparative analysis of targeted novel therapies in relapsed, refractory chronic lymphocytic leukaemia. Haematologica. 2021;106(1):284-287. doi:10.3324/haematol.2019.241539 32079693 PMC7776352

[4] Centers for Medicare & Medicaid Services. Medicare part D prescribers by geography and drug dataset February 2022. Accessed October 1, 2022. https://data.cms.gov/provider-summary-by-type-of-service/medicare-part-d-prescribers/medicare-part-d-prescribers-by-geography-and-drug

[5] Sarpatwari A, DiBello J, Zakarian M, Najafzadeh M, Kesselheim AS. Competition and price among brand-name drugs in the same class: a systematic review of the evidence. PLoS Med. 2019;16(7):e1002872. doi:10.1371/journal.pmed.1002872 31361747 PMC6667132

[6] Cole AL, Dusetzina SB. Generic price competition for specialty drugs: too little, too late? Health Aff (Millwood). 2018;37(5):738-742. doi:10.1377/hlthaff.2017.1684 29733710

