# Supplementary material for: Trends in Medicare Spending on Oral Drugs for Chronic Lymphocytic Leukemia From 2014 to 2020
Source: JAMA Netw Open. 2023 Apr 7;6(4):e237467. doi: 10.1001/jamanetworkopen.2023.7467 (PMC10082405; doi:10.1001/jamanetworkopen.2023.7467)
Supplement: Supplement. — Data Sharing Statement [file jamanetwopen-e237467-s001.pdf]

Data Sharing Statement

Cliff. Trends in Medicare Spending on Oral Drugs for Chronic Lymphocytic Leukemia From 2014 to 2020. *JAMA Netw Open*. Published April 07, 2023.
doi:10.1001/jamanetworkopen.2023.7467

Data
Data available: Yes
Data types: Other (please specify)
Additional Information: Publicly available data
How to access data: CMMS website
When available: With publication

Supporting Documents
Document types: None

Additional Information
Who can access the data: Anyone - data already publicly available
Types of analyses: Any reasonable purpose
Mechanisms of data availability: Already publicly available
